# Proteomic analysis of the effect of high-fat-diet and voluntary physical activity on mouse liver

**DOI:** 10.1371/journal.pone.0273049

**Published:** 2022-08-18

**Authors:** Byunghun So, Li Li Ji, Saba Imdad, Chounghun Kang

**Affiliations:** 1 Molecular Metabolism in Health & Disease, Exercise Physiology Laboratory, Inha University, Incheon, Republic of Korea; 2 Laboratory of Physiological Hygiene and Exercise Science, School of Kinesiology, University of Minnesota Twin Cities, Minneapolis, MN, United States of America; 3 Department of Biomedical Laboratory Science, College of Health Science, Cheongju University, Cheongju, Republic of Korea; 4 Department of Physical Education, College of Education, Inha University, Incheon, Republic of Korea; Universidade do Estado do Rio de Janeiro, BRAZIL

## Abstract

Nonalcoholic fatty liver disease (NALFD), characterized by an abnormal accumulation of triglycerides in hepatocytes, is closely linked to insulin resistance, metabolic syndrome, and changes in lipogenesis in the liver. The accumulation of hepatic lipids can lead to a range of pathologies from mild steatosis to severe cirrhosis. Endurance exercise is known to ameliorate the adverse health effects of NAFLD. Therefore, we aimed to investigate the effect of voluntary wheel running (VWR) on the metabolic changes in the livers of high-fat diet (HFD)-induced NAFLD mice and used LC-MS/MS (Liquid chromatography–mass spectrometry) to determine whether the tested intervention affected the protein expression profiles of the mouse livers. Male C57BL/6 mice were randomly divided into three groups: control (CON), high-fat diet sedentary group (HFD), high-fat diet VWR group (HFX). HFX group performed voluntary wheel running into individually cages, given a high-fat diet for 12 weeks. Food consumption, body weight, and running distance were measured every week. Using 2D (2-dimensional)-gel electrophoresis, we detected and quantitatively analyzed the protein expression with >2.0-fold change in the livers of HFD-fed mice, HFD-fed exercise (HFX) mice, and chow-fed mice. Body weight was significantly increased in HFD compared to CON (P < 0.05). The 2D-gel electrophoresis analysis indicated that there was a difference between CON and HFD groups, showing 31 increased and 27 decreased spots in the total 302 paired spots in the HFD group compared to CON. The analysis showed 43 increased and 17 decreased spots in the total 258 spots in the HFX group compared to CON. Moreover, 12 weeks of VWR showed an increase of 35 and a decrease of 8 spots in a total of 264 paired spots between HFD and HFX. LC-MS/MS of HFD group revealed that proteins involved in ketogenesis, lipid metabolism, and the metabolism of drugs and xenobiotics were upregulated, whereas detoxifying proteins, mitochondrial precursors, transport proteins, proteasomes, and proteins involved in amino acid metabolism were downregulated. On the other hand, VWR counteracted the protein expression profile of HFD-fed mice by upregulating molecular chaperones, gluconeogenesis-, detoxification-, proteasome-, and energy metabolism-related proteins. This study provided a molecular understanding of the HFD- and exercise-induced protein marker expression and presented the beneficial effects of exercise during pathophysiological conditions.

## Introduction

Nonalcoholic fatty liver disease (NAFLD) is a condition in which excessive triglyceride (TG) accumulation in hepatocytes results in steatosis and inflammation [1, 2]. The pathophysiology of NAFLD ranges from nonalcoholic steatohepatitis, which increases the inflammatory response and hepatocyte fibrosis to hepatic cirrhosis and hepatocellular carcinoma [3, 4]. A growing number of studies suggest that NAFLD is associated with metabolic syndrome-related diseases [5], such as type 2 diabetes, obesity, and dyslipidemia, and significantly increases the risk of cardiovascular disease [6]. Excessive caloric intake, including a high-fat diet (HFD) and inactivity, plays a critical role in the development of NAFLD [7]. In addition, HFD-induced metabolic disorders are intimately linked to and promoted by high availability of plasma free fatty acids in the liver [8].

Above all, early NAFLD treatment is essential to prevent severe liver disease, but so far, there is no approved medication available for NAFLD. However, numerous studies have attempted to explore and find an effective solution to attenuate NAFLD development, including calorie restriction and physical exercise [9–11]. Regular exercise is an effective regimen for the prevention and treatment of metabolic disorders. The principal advantage of exercise in NAFLD is improved insulin sensitivity and lipid profile, and a reduction in the fat accumulation in the liver [11].

Several studies have been conducted to investigate the beneficial effects of exercise using HFD models. A comparison of the effects of forced treadmill and voluntary wheel running exercise in a mouse model showed a similar change of physiologic effects, such as weight, fat mass, and mitochondrial biogenesis markers [12]. However, it is commonly known that forced treadmill exercise can be adjusted in duration and intensity, while VWR can help to become habitual with continued access to participate in aerobic exercise [13]. Forced treadmill exercise was demonstrated to enhance fatty acid oxidation by improving mitochondrial function and suppressing lipogenesis expression in the liver [14, 15]. In addition, endurance exercise was reported to ameliorate HFD-induced mitochondrial function in the liver, such as that related to the mitochondrial transmembrane electric potential and fat oxidation [16] Several studies showed that VWR can protect against inflammatory markers in adipose tissue and ameliorate insulin sensitivity in diet-induced obese mouse model [17–19]. Ghareghani et al. demonstrated that aerobic endurance training can reduce lipogenesis by miR-33-mediated autophagy, which regulates cholesterol homeostasis in an HFD model [20]. To date, despite the accumulating evidence of the positive effects of exercise in the HFD model, studies to find exercise-related protein marker profile are needed for further research.

High-throughput techniques can fill the knowledge gap regarding the elucidation of the underlying mechanism of exercise intervention in metabolic disorders at the level of individual genes or translational proteins [21, 22]. Several studies have suggested that proteomics is a powerful tool for assessing quantitative protein expression in specific organs [22, 23]. Previous studies have demonstrated the impact of HFD on mouse liver gene expression to quantitatively screen and identify protein expression patterns using MS/MS analysis [24, 25].

In the present study, we investigated the protein expression profiles in the liver of HFD-fed mice under sedentary and aerobic exercise conditions to understand the underlying molecular mechanisms associated with the protective effects of exercise. We screened for proteins involved in *de novo* synthesis, energy metabolism, and mitochondrial function in the HFD mouse model using LC-MS/MS.

## Materials and methods

### Experimental animals and sample preparation

Five-week-old mice (30 male, C57BL/6, DBL Co., Korea) were purchased and randomly assigned to three groups: CON, control group (n = 9); HFD, high-fat diet sedentary group (n = 9); HFX, high-fat diet VWR group (n = 12). The CON group was fed a chow diet (18% protein, 5% fat, 5% fiber, 5% ash, RodFeed, DBL, Co., Korea) and the HFD group was fed a high-fat diet (34.9% fat by weight, providing 60% calories from fat, D12492, Research diets, Co., USA) for 12 weeks. The animals were maintained in a room with constant temperature of 22 ± 1°C and humidity-controlled environment 55 ± 10% with a 12-light/12-h dark cycle and free access to food and water. Body weight and food intake of the animals were measured weekly. The institutional animal care and use committee of Chungbuk National University reviewed and approved the study design before the animal experiments were conducted (CBNUA-1214-18-01). The procedures for the handling and care of the animals adhered to the guidelines that comply with the current international laws and policies (NIH Guide for the Care and Use of Laboratory Animals, NIH Publication No. 85–23, 1985, revised 1996) [26]. All experiments were conducted to minimize the number of animals used and the possible suffering/pain that may be caused by any of the experimental procedures used in the present study.

The mice were anesthetized with 2.5% flow rate of isoflurane (Sigma-Aldrich, USA) using Multiplus-MEVD anesthesia machine with ventilator (Royal Medical Co., Korea), and were sacrificed immediately post anesthesia by cervical dislocation. Frozen liver tissues (200 mg) were solubilized in 1.0 ml of lysis buffer consisting of 7 M urea, 2M thiourea, 4% w/v CHAPS, 100 mM dithiolerythritol DTE, 40 mM Tris, 2% v/v pH 3–10 Bio-Lytes (Bio-rad, Hercules, CA, USA) and a trace of bromphenole blue. Solubilization was aided by tip-probe sonication for 4 x 30s with 2min on ice between each round of sonication. Samples were centrifuged at 12000 g at 4°C for 1 h. The supernatant was transferred into new tubes, and then 150 U of endonuclease (Sigma, St. Louis, MO, USA) was added. Protein samples were stored at −80°C until use.

### Voluntary wheel running

The HFX group was individually housed, and a running wheel (Lafayette Instrument Company, USA) was placed in each cage for 12 weeks. Running activities such as distance (meter) and velocity (meter per minute) were recorded in a 10-min time interval using a computerized system (Lafayette instrument computerized animal wheel monitoring system; Lafayette Instrument Co., USA).

### 2D-Polyacrylamide gel electrophoresis

2D-Polyacrylamide gel electrophoresis (PAGE) was performed as described previously [27, 28]. Aliquots in sample buffer (7 M urea, 2M thiourea, 4.5% CHAPS, 100 mM DTE, 40 mM Tris, pH 8.8) were applied to the immobilized non-linear gradient (pH 3–10) strips (Amersham Biosciences, Uppsala, Sweden). Isoelectric focusing was performed at 80,000 Vh (volt-hour). The second dimension was analyzed on 9%–16% linear gradient polyacrylamide gels (18 cm × 20 cm × 1.5 mm) at a constant 40 mA per gel for approximately 5 h. After protein fixation in 40% methanol and 5% phosphoric acid for 1 h, the gels were stained with Coomassie Brilliant Blue G-250 for 12 h. The gels were then destained with H_2_O, scanned in a Bio-Rad (Richmond, CA) GS710 densitometer, and converted into electronic files, which were then analyzed with Image Master Platinum 5.0 (Amersham Biosciences).

### LC-MS/MS for peptide analysis

Nano LC-MS/MS analysis was performed with an Easy n-LC (Thermo Fisher San Jose, CA, USA) and an LTQ Orbitrap XL mass spectrometer (Thermo Fisher, San Jose, CA, USA) equipped with a nano-electrospray source. Samples were separated on a C18 nanopore column (150 mm × 0.1 mm, 3 μm pore size; Agilent). Mobile phase A for LC separation was 0.1% formic acid and 3% acetonitrile in deionized water, and mobile phase B was 0.1% formic acid in acetonitrile [28]. The chromatography gradient was designed for a linear increase from 0% B to 60% B in 9 min, 60% B to 90% B in 1 min, and 3% B in 5 min. The flow rate was maintained at 1800 nL/min.

Mass spectra were acquired using data-dependent acquisition with a full mass scan (380–1700 *m/z*) followed by 10 MS/MS scans. For MS1 full scans, the orbitrap resolution was 15,000, and the automatic gain control (AGC) was 2×10^5^. For MS/MS in the LTQ, the AGC was 1×10^4^.

### Database search

The mascot algorithm (Matrixscience, USA) was used to identify peptide sequences present in a protein sequence database. Database search criteria were taxonomy: *Mus musculus*, fixed modification; carbamidomethylated at cysteine residues; variable modification; oxidized at methionine residues, maximum allowed missed cleavage, 2; MS tolerance, 10 ppm; MS/MS tolerance, 0.8 Da. The peptides were filtered using a significance threshold of P <0.05.

### Statistical and network analysis

Experimental data were expressed as mean ± standard error of the mean (SEM), and the effect of time and exercise on physiological parameters such as weight and food intake were analyzed using two-way ANOVA with Tukey-Kramer post-hoc test with a standard significance threshold (P < 0.05). GraphPad Prism 8.0 (CA, USA) was used for statistical analysis. To analyze the interactions between HFD-dysregulated proteins and HFX-upregulated proteins found in this study, we uploaded differentially regulated metabolism-related proteins, with a significance of >2.0-fold, to the STRING database (Search Tool for the Retrieval of Interacting Genes/Proteins, version 11.0) to search for protein–protein interactions.

## Results

### Impact of VWR on body weight and food uptake

**Fig 1:**
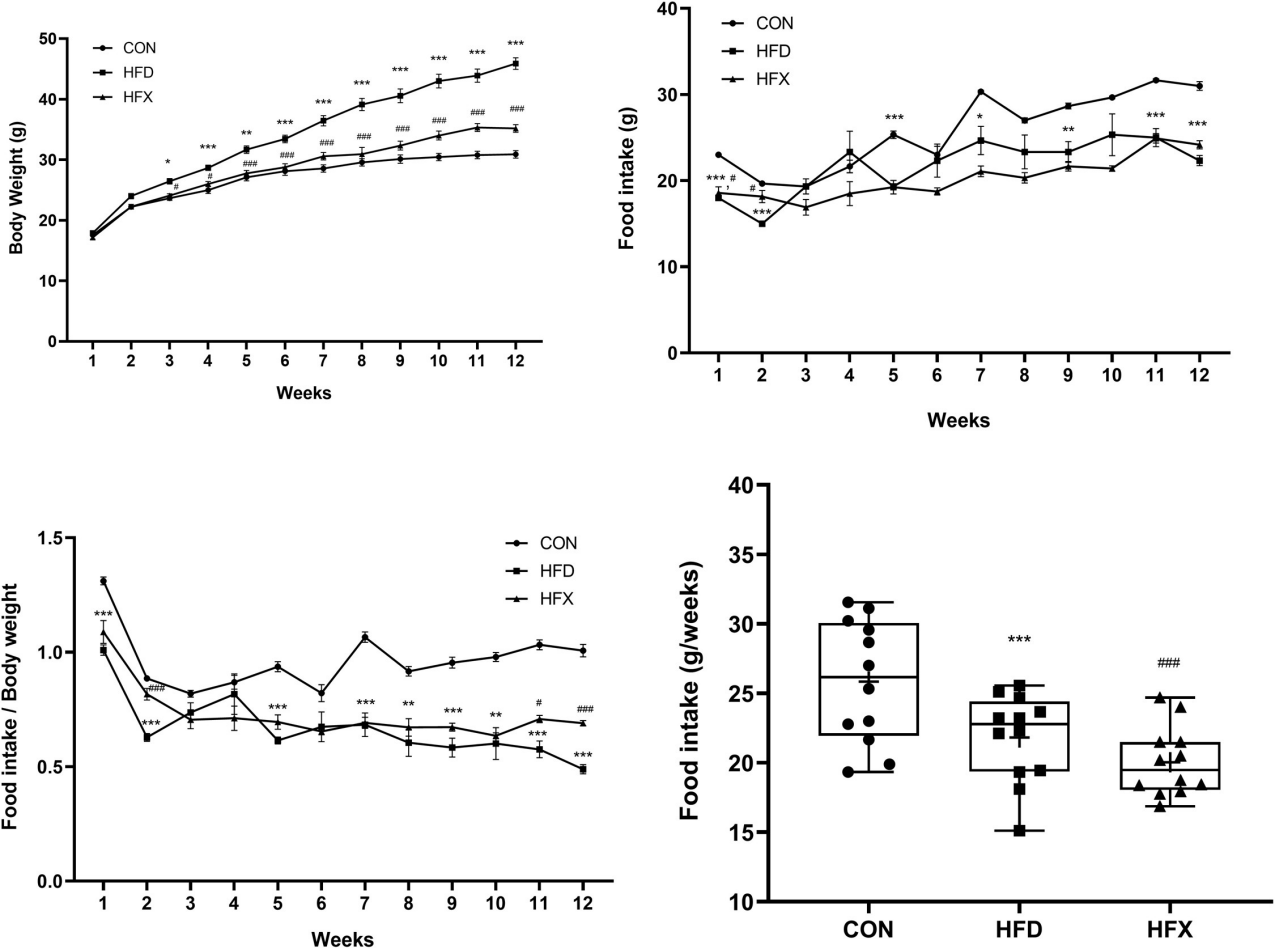
The effects of a high-fat diet and voluntary wheel running (VWR) on body weight, food intake. C57BL/6 mice were fed a high-fat diet (HFD), a high-fat fed with VWR (HFX), and corresponding control diet (CON) for 12 weeks. (A) The body weight curve of the CON, HFD, and HFX group was monitored. (B) Weekly food intake of the CON, HFD, and HFX group for 12 weeks. (C) The ratio of weekly average food intake to body weight for all experimental groups. (D) Distribution of food intake for all experimental groups. The data represent means ± SEM of (A-D) n = 9, the control group (CON); n = 9, the high-fat diet group (HFD); n = 12, the high-fat diet VWR group (HFX). **P<0.01, ***P<0.001 compared with CON and ^##^P<0.01, ^###^P<0.001 compared with HFD, using two-way ANOVA with Tukey-Kramer post-hoc test with a standard significance threshold (P<0.05) (A-D).

Fig 1A demonstrate the weekly body weight for each group. HFD induced a significant weight gain from 3-week to 12-week period in the HFD group compared to the CON group (P < 0.001). High fat diet had a very significant effect on the body weights [F(22, 324) = 12.88, P < 0.001]. However, voluntary wheel running for 12 weeks prevented weight gain in the HFX group (P < 0.001). The food intake was higher in the CON group than in the HFD group [F(22, 324) = 3.844, P < 0.001; Fig 1B)]. In contrast, there was no difference in the food consumption between the HFD and HFX groups after 12 weeks. When the body weights of the mice were normalized, we found that the food intake of mice was significantly higher in the CON group than the other groups. And HFX group had a higher food intake than HFD group (P < 0.001, Fig 1C). Moreover, cumulative food intake was significantly higher in the CON group than HFD group (P < 0.001), while the HFX group had a decreased cumulative intake as compared to HFD group (P < 0.001, Fig 1D). In addition, VWR showed a gradual decrease in the running distance over time in the HFX group, at the individual level and a large variation was observed in the total running distance ranging from about 113 km to 247 km in the HFX group (S1 Fig).

### 2D-PAGE analysis

**Fig 2:**
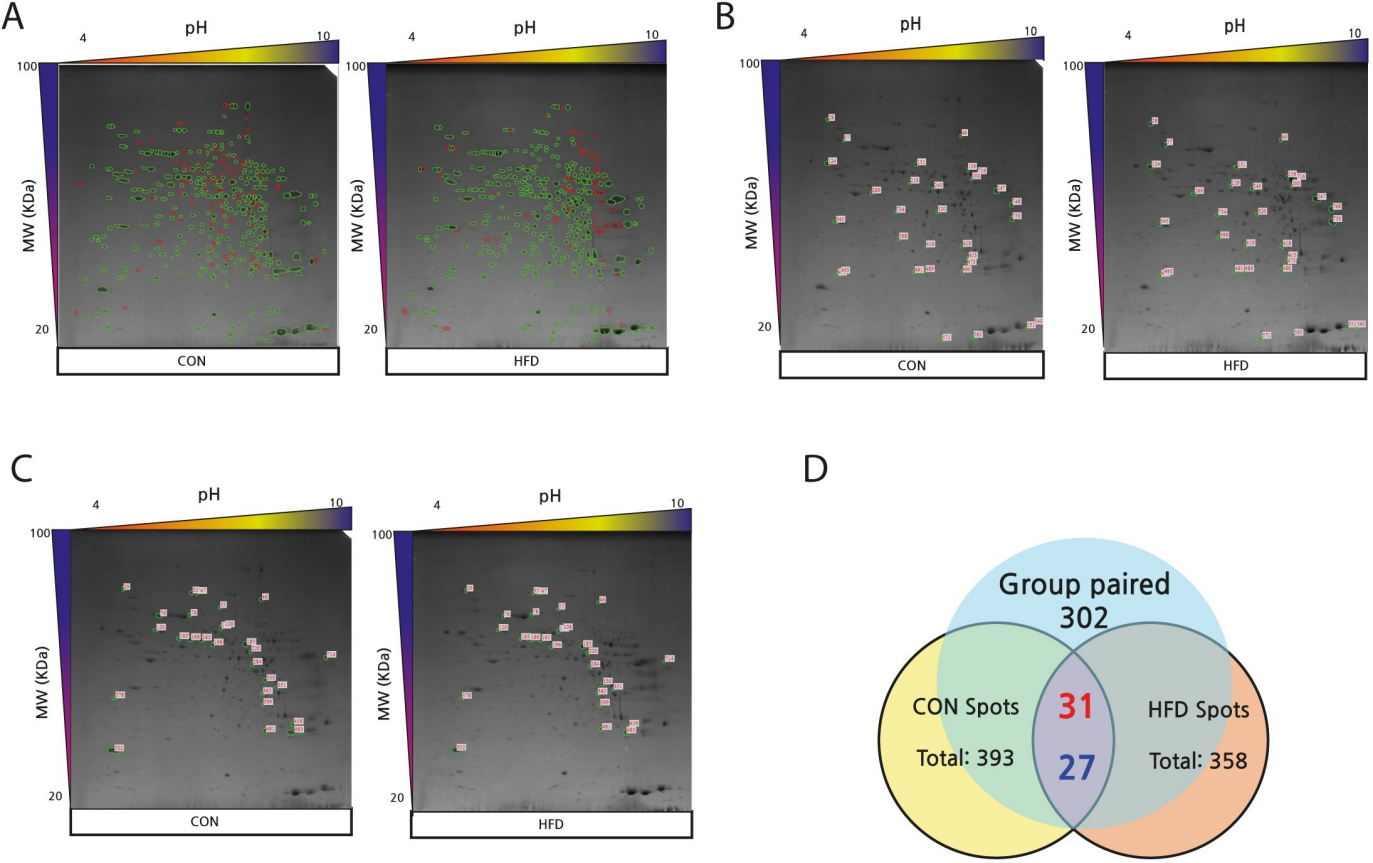
2D-PAGE images and Venn diagram showing differential protein expression (>2-fold) between CON and HFD groups (A-D). (A) The images of 2D-PAGE show green circles indicating group paired spots, and red circles indicating group non-paired spots. Green circles indicate group paired spots, and orange circles indicate group non-paired spots. The images of the 2D-PAGE show an increase of 31 spots (B) and a decrease of 27 spots (C) among the 302 paired spots in HFD compared to CON. The differentially expressed spots showed a difference of >2.0-fold. (D) The numbers in red denote upregulated spots and the numbers in blue indicate downregulated spots, in the Venn diagram.

2D-PAGE analysis of the mouse liver showed a total of 393 and 358 protein spots for the CON and HFD groups, respectively, with 147 non-paired spots between the two groups. There were 302 paired protein spots between the CON and HFD groups, which were used for further analysis. Among the 302 paired spots, 31 protein spots were found to be upregulated, while 27 spots were downregulated in the HFD group compared with the CON group (Fig 2A–2D).

**Fig 3:**
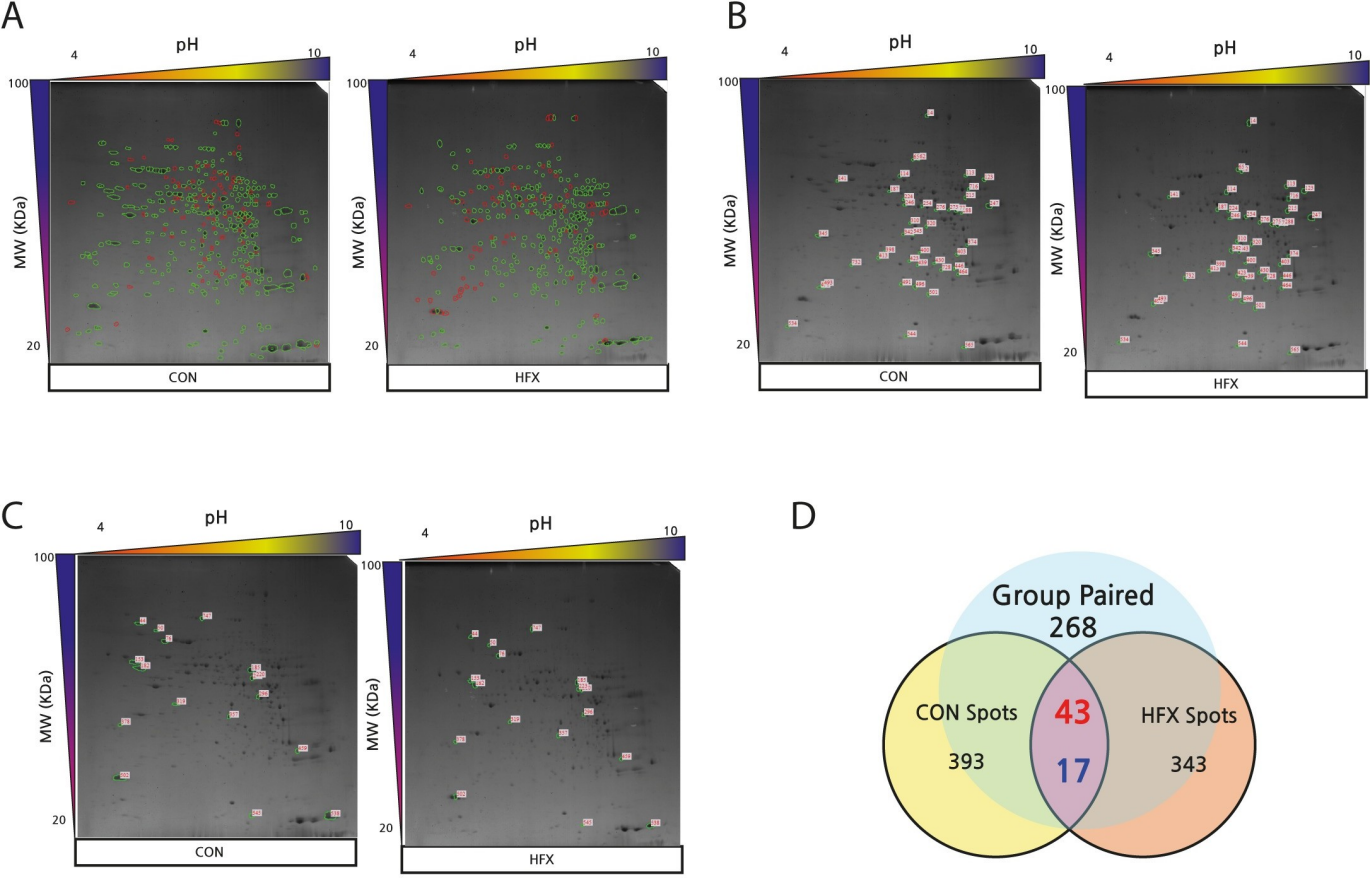
2D-PAGE images and Venn diagram showing differential protein expression (>2-fold) between CON and HFX (A-D). (A) The images of 2D-PAGE show green circles which indicate group paired spots, and red circles that indicate group non-paired spots. Green circles indicate group paired spots, and orange circles indicate group non-paired spots. The images of the 2D-PAGE show an increase of 43 spots (B) and a decrease of 17 spots (C) among the 268 paired spots in HFD compared to CON. The differentially expressed spots showed a difference of >2.0-fold. (D) The numbers in red denote upregulated spots and the numbers in blue indicate downregulated spots, in the Venn diagram.

**Fig 4:**
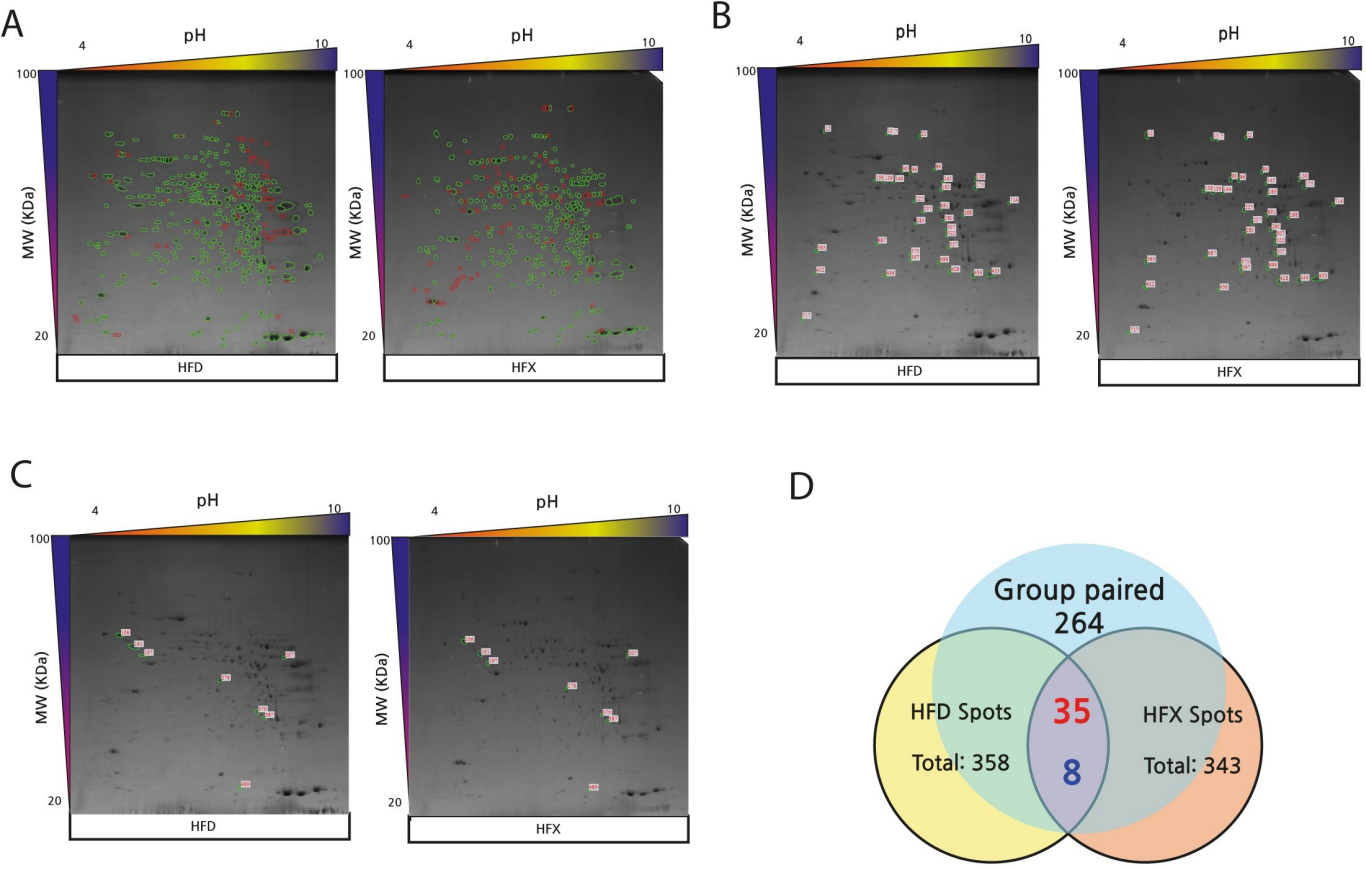
2D-PAGE images and Venn diagram showing differential protein expression (>2-fold) between HFD and HFX (A-D). (A) The images of 2D-PAGE show green circles that indicate group paired spots, and red circles that indicate group non-paired spots. Green circles indicate group paired spots, and orange circles indicate group non-paired spots. The images of the 2D-PAGE show an increase of 35 spots (B) and a decrease of 8 spots (C) among the 264 paired spots in HFD compared to CON. The differentially expressed spots showed a difference of >2.0-fold. (D) The numbers in red denote upregulated spots and the numbers in blue indicate downregulated spots, in the Venn diagram.

Among the total 343 protein spots detected in the HFX group, 268 were paired spots between CON and HFX, while the number of non-paired spots was 200. Analysis of the 268 paired spots revealed 43 upregulated and 17 downregulated proteins in the HFX group (Fig 3A–3D). Among the 264 paired spots found between the HFD and HFX groups, 35 protein spots corresponded with upregulated, while 8 spots corresponded with downregulated proteins in the HFX group. A total of 173 non-paired protein spots were detected (Fig 4A–4D). The differentially expressed proteins showed a difference of >2.0-fold.

### Proteomic analysis of mouse liver

**Fig 5:**
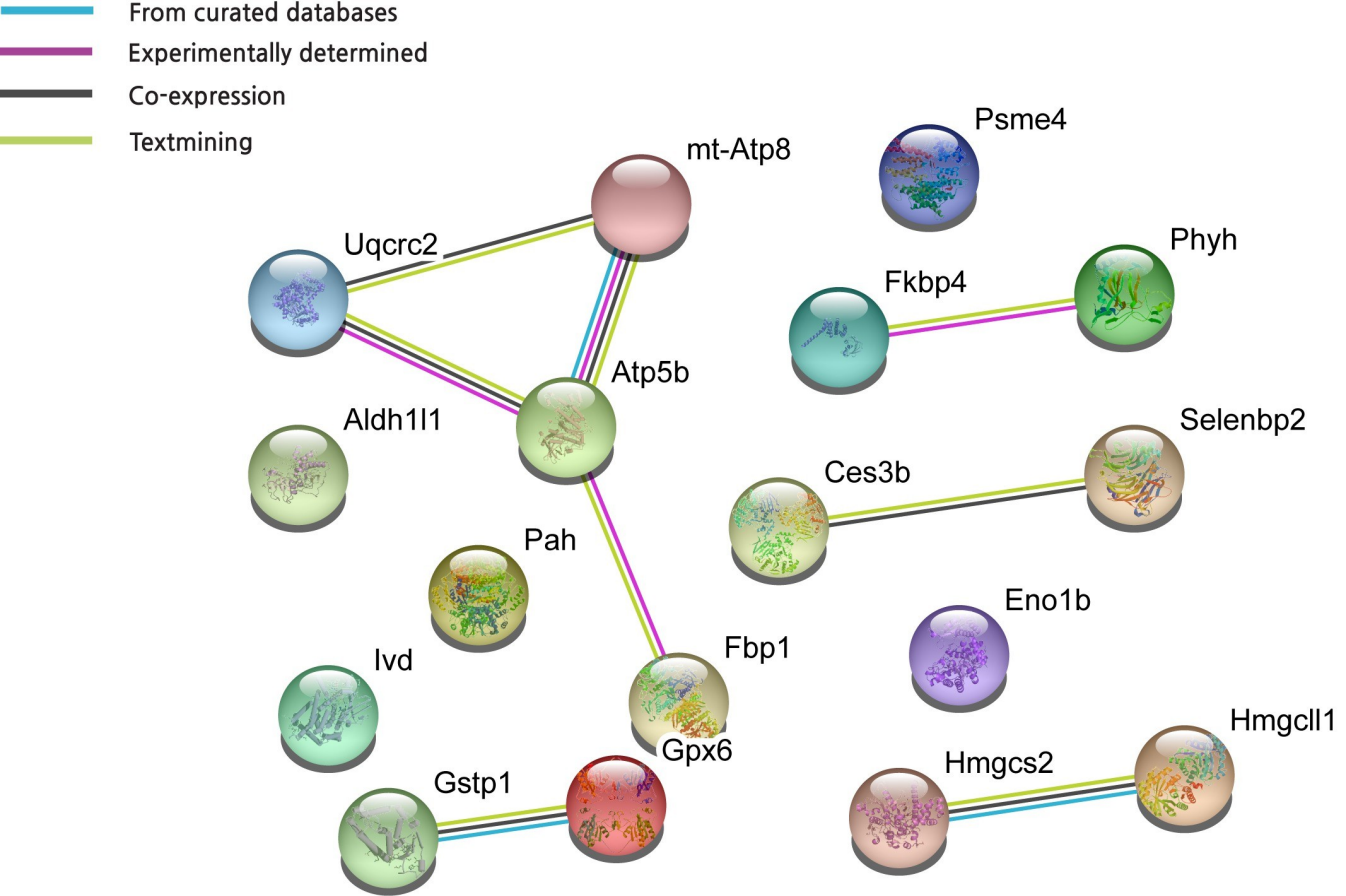
Protein-protein interaction scheme generated from differentially regulated protein upon HFD, and exercise focused on metabolism using STRING (version 11.0).

The proteomic analysis of the mouse livers using 2D-PAGE followed by protein identification by LC-MS/MS revealed 35 protein spots whose expression levels were changed by > 2.0-fold with significant alterations (P<0.05) in the HFD vs. CON (Table 1), HFX vs. CON groups (Table 2) and the HFX vs. HFD groups (Table 3). According to their functional properties, the identified proteins were grouped into the following nine categories:

1. Proteins involved in carbohydrate, lipid, and energy metabolism, such as fructose-1,6- bisphosphate 1 (Fbp1), ATP synthase (mt-Atp8), ATP5b protein (Atp5b), and enolase 1 B (Eno1b)
2. Proteins involved in amino acid metabolism, such as phenylalanine hydroxylase (Pah), isovaleryl-CoA dehydrogenase (Ivd), and carboxylesterase 3 B (Ces3b)
3. Mitochondrial precursors, such as isovaleryl-CoA dehydrogenase (Ivd), cytochrome b-c1 complex subunit 2 (Uqcrc2), 3-hydroxy-3-methylglutarly-coenzyme synthase 2 (Hmgcs2), 3-hydroxy-3-methlglutarly-CoA synthase (Hmgcs2), and 3-hydroxy-3-methlglutarly-CoA lyase (Hmgcl1)
4. Molecular chaperones, such as heat shock protein 90-beta membrane 1 (Fkbp4)
5. Proteins involved in detoxification, such as glutathione S-transferases (Gstp1) and glutathione peroxidase 6 (Gpx6), the crystal structure of a murine alpha-class glutathione S-transferase (1GUK_A)
6. Protein transporters, such as selenium-binding protein (Selenbp2)
7. Proteolysis proteins, such proteasome activator subunit 4 (Psme4)
8. Enzymes, such as Aldh1l1 (Aldh1l1)
9. Unknown functional proteins, such as the mCG8752 isoform CRA_c, mCG8752 isoform CRA_b, and mCG129115 (Fig 5).

**Table 1:** Liver proteins differentially expressed in the HFD vs. CON.

Accession Number	Symbols	Protein name	Mass	Total Mascot score	Matched peptide	Sequences	Calculated PI	Sequence Coverage	emPAI	Fold change (HFD/CON)	Spot number
AAH48380.1	Ces3b	Carboxylesterase 3B	63790	52	1(1)	1(1)	5.79	1	0.11	1.2	114
NP_062287.1	Selenbp2	selenium-binding protein 2	53165	933	97(97)	15(15)	5.78	31	3.13	-3.4	165
NP_062287.1	Selenbp2	selenium-binding protein 2	53165	1075	90(90)	18(18)	5.78	31	4.92	-2.5	166
NP_058054.2	Hmgcs2	ATP synthase subunit beta	56265	1464	253(253)	19(19)	5.19	40	8.62	1.1	182
AAH37127.1	Atp5b	Atp5b protein	56632	597	28(28)	10(10)	5.24	23	1.73	-1.2	215
EDL38956.1	Hmgcs2	3-hydroxy-3-methylglutaryl-Coenzyme A synthase 2	59142	509	13(13)	9(9)	8.36	14	1.10	1.9	217
EDL21456.1	Pah	phenylalanine hydroxylase	50265	506	22(22)	11(11)	5.91	20	1.84	1.2	224
NP_001020559.1	Eno1b	enolase 1B	47453	1129	51(51)	20(20)	6.37	31	7.87	-1.4	254
NP_062800.1	Ivd	isovaleryl-CoA dehydrogenase	44695	626	30(30)	11(11)	8.53	23	3.56	-2.2	284
AAB03107.1	Hmgcll1	3-hydroxy-3-methylglutaryl-CoA lyase	34641	44	1(1)	1(1)	8.70	2	0.15	-2.9	365
EDL26822.1	Psme4	proteasome	31081	139	2(2)	2(2)	8.80	6	0.37	-1.3	423
EDL01226.1	mCG8752	mCG8752, isoform CRA_b	31728	262	10(10)	5(5)	6.47	15	1.10	-2.1	399
NP_038569.1	Gstp1	glutathione S-transferase P 1	23765	209	4(4)	3(3)	7.68	18	1.20	-3.9	483
1GUK_A	1GUK_A	Chain A, Crystal Structure of Murine Alpha-Class Gsta4-4	25559	534	23(23)	11(11)	6.77	38	6.54	-3.7	481
EDL31590.1	mt-Atp8	ATP synthase	17589	158	6(6)	2(2)	5.01	16	0.70	-1.3	534

**Table 2:** Liver proteins differentially expressed in the HFX vs. CON.

Accession Number	Symbols	Protein name	Mass	Total Mascot score	Matched peptide	Sequences	Calculated PI	Sequence Coverage	emPAI	Fold change (HFX/CON)	Spot number
AAH48380.1	Ces3b	Carboxylesterase 3B	63790	52	1(1)	1(1)	5.79	1	0.11	4.2	114
NP_062287.1	Selenbp2	selenium-binding protein 2	53165	933	97(97)	15(15)	5.78	31	3.13	1.1	165
NP_062287.1	Selenbp2	selenium-binding protein 2	53165	1075	90(90)	18(18)	5.78	31	4.92	1.2	166
NP_058054.2	Hmgcs2	ATP synthase subunit beta	56265	1464	253(253)	19(19)	5.19	40	8.62	-2.1	182
AAH37127.1	Atp5b	Atp5b protein	56632	597	28(28)	10(10)	5.24	23	1.73	2.6	215
EDL38956.1	Hmgcs2	3-hydroxy-3-methylglutaryl-Coenzyme A synthase 2	59142	509	13(13)	9(9)	8.36	14	1.10	-1.9	217
EDL21456.1	Pah	phenylalanine hydroxylase	50265	506	22(22)	11(11)	5.91	20	1.84	4.9	224
NP_001020559.1	Eno1b	enolase 1B	47453	1129	51(51)	20(20)	6.37	31	7.87	2.7	254
NP_062800.1	Ivd	isovaleryl-CoA dehydrogenase	44695	626	30(30)	11(11)	8.53	23	3.56	1.1	284
AAB03107.1	Hmgcll1	3-hydroxy-3-methylglutaryl-CoA lyase	34641	44	1(1)	1(1)	8.70	2	0.15	1.6	365
EDL26822.1	Psme4	proteasome	31081	139	2(2)	2(2)	8.80	6	0.37	2.5	423
EDL01226.1	mCG8752	mCG8752, isoform CRA_b	31728	262	10(10)	5(5)	6.47	15	1.10	1.2	399
NP_038569.1	Gstp1	glutathione S-transferase P 1	23765	209	4(4)	3(3)	7.68	18	1.20	-1.8	483
1GUK_A	1GUK_A	Chain A, Crystal Structure of Murine Alpha-Class Gsta4-4	25559	534	23(23)	11(11)	6.77	38	6.54	1.2	481
EDL31590.1	mt-Atp8	ATP synthase	17589	158	6(6)	2(2)	5.01	16	0.70	2.3	534

**Table 3:** Liver proteins differentially expressed in the HFX vs. HFD.

Accession Number	Symbols	Protein name	Mass	Total Mascot score	Matched peptide	Sequences	Calculated PI	Sequence Coverage	emPAI	Fold change (HFX/HFD)	Spot number
AAH10445.1	Fkbp4	Hsp90b1 Heat shock protein 90, beta (Grp94), member 1	92717	2808	87(87)	55(55)	4.74	42	21.44	5.7	12
AAH24055.1	Aldh1l1	Aldh1l1 protein	99527	1323	42(42)	26(26)	5.69	25	3.16	3.7	15
AAH48380.1	Ces3b	Carboxylesterase 3B	63790	52	1(1)	1(1)	5.79	1	0.11	4.2	90
NP_062287.1	Selenbp2	selenium-binding protein 2	53165	752	43(43)	12(12)	5.78	23	2.46	3.8	138
NP_062287.1	Selenbp2	selenium-binding protein 2	53165	1432	97(97)	23(23)	5.78	37	9.04	3.2	139
NP_062287.1	Selenbp2	selenium-binding protein 2	53165	1594	127(127)	23(23)	5.78	47	8.55	3.8	144
NP_062287.1	Selenbp2	selenium-binding protein 2	53165	455	18(18)	8(8)	5.78	17	1.03	4.6	145
NP_062287.1	Selenbp2	selenium-binding protein 2	53165	212	5(5)	4(4)	5.78	7	0.43	3.3	183
NP_032282.2	Hmgcs2	hydroxymethylglutaryl-CoA synthase	57300	1115	120(120)	19(19)	8.65	27	4.64	-2.5	207
NP_032282.2	Hmgcs2	hydroxymethylglutaryl-CoA synthase	57300	201	7(7)	4(4)	8.65	7	0.39	2.5	370
EDL01201.1	mCG129115	mCG129115	52628	144	3(3)	3(3)	4.95	5	0.31	-2.9	197
CAA27558.1	Gpx6	glutathione peroxidase	22504	335	10(10)	6(6)	6.74	32	3.28	2.7	225
NP_062800.1	Ivd	isovaleryl-CoA dehydrogenase	46695	933	65(65)	17(17)	8.53	29	7.36	2.5	691
NP_062268.1	Fbp1	fructose-1,6-bisphosphatase 1	37288	1185	97(97)	18(18)	6.15	38	11.47	5.2	285
BAA19003.1	Phyh	LN1	39053	542	23(23)	12(12)	7.64	26	6.97	4.6	322
EDL01227.1	mCG8752	mCG8752, isoform CRA_c	50255	802	59(59)	13(13)	6.02	26	2.72	2.6	357
EDL26822.1	Psme4	proteasome	31081	139	2(2)	2(2)	8.80	6	0.37	2.5	375
NP_038569.1	Gstp1	glutathione S-transferase P 1	23765	345	19(19)	5(5)	7.68	28	4.90	4.4	428
NP_080175.1	Uqcrc2	cytochrome b-c1 complex subunit 2	48262	971	34(34)	16(16)	9.26	30	5.09	3.4	714
EDL31590.1	mt-Atp8	ATP synthase	17589	158	6(6)	2(2)	5.01	16	0.70	2.3	717

### Impact of HFD on biochemical pathways

**Fig 6:**
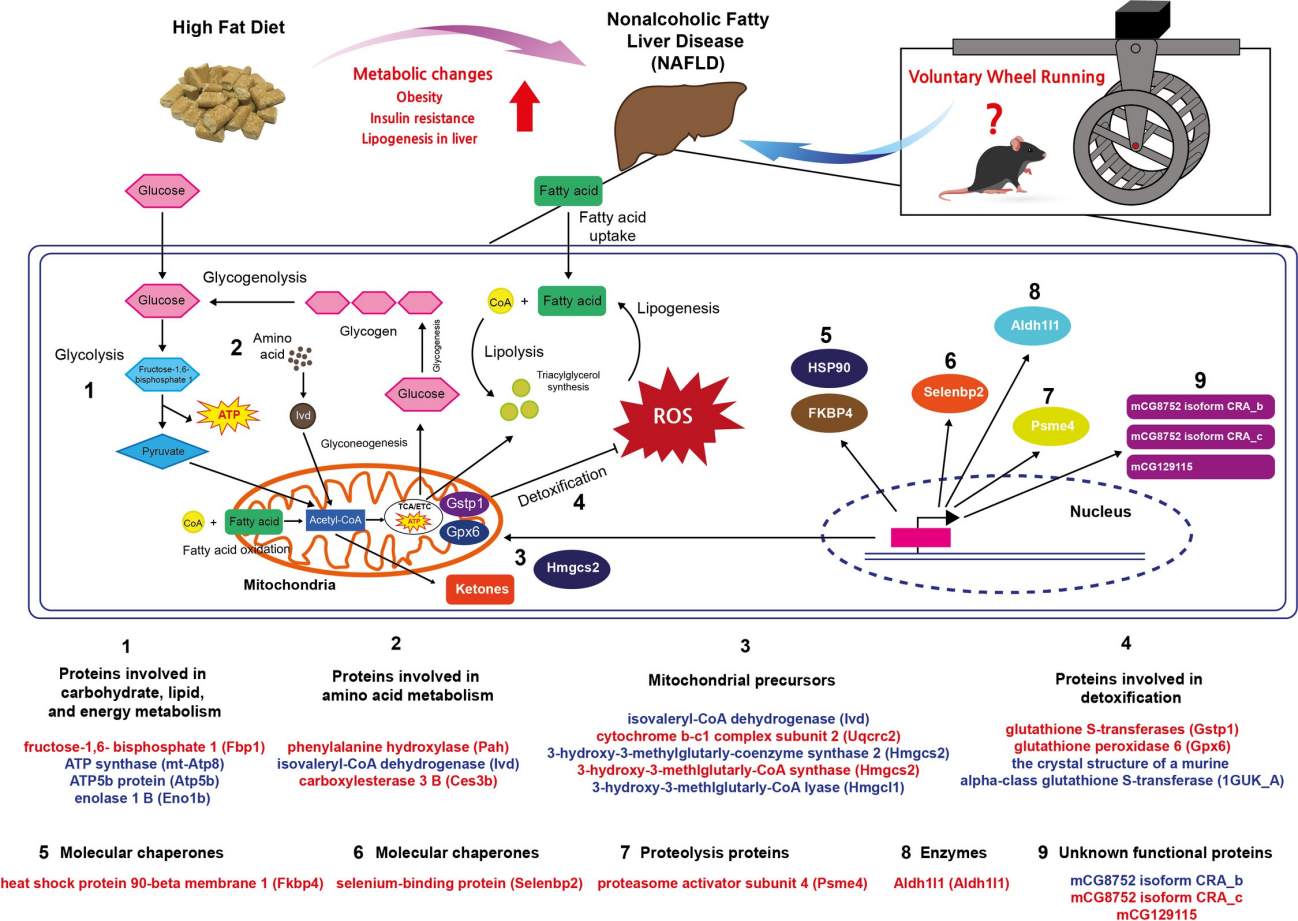
Summary of differentially expressed liver proteins from a high-fat diet and 12 weeks of voluntary wheel running intervention. Interpretation of proteomic analysis results by LC-MS/MS based on enriched functional annotation of proteins present at higher or lower levels in HFX compared to HFD. Proteins were grouped according to their functional properties into nine categories, as shown in the figure. ATP, adenosine triphosphate; TCA, tricarboxylic acid cycle; ETC, electron transport chain; ROS, reactive oxygen species; CoA, coenzyme A. Red color denotes upregulated protein expression in the HFX group compared to the HFD group and blue color indicates downregulated protein expression in the HFD group compared to the CON group.

To determine a functional network or interaction between the HFD and HFX protein regulation profiles, we performed four functional association analyses using the STRING database (version 11.0, https://string-db.org) on 35 protein spots. The network showed that database (light blue lines) and co-expression (black lines) associations are generated from a list of significant protein interaction groups gathered from curated databases. The experimental association (pink lines) was extracted from a list of significant protein interaction datasets gathered from other protein–protein interaction databases, such as IMEx and MIntAct. The text mining associations (light green lines) were extracted from published scientific literature. The majority of these 28 differentially regulated proteins showed protein–protein interactions (PPI) in all four association algorithms. In the protein network, mt-Atp8, Uqcrc2, and Atp5b, which were related to electron transport chain (ETC), exhibited more interactions than the other proteins. Interestingly, those three proteins mentioned above were also downregulated in the HFD but were upregulated by the physical activity in the HFX group (Fig 6).

## Discussion

High-fat diet induces the development of NAFLD, the most widespread chronic liver disease in many regions of the globe, especially in Western countries [29, 30]. NAFLD is also considered an independent predictor of cardiovascular diseases and increases the risk of chronic kidney disease [31]. However, the current therapeutic outcomes for dealing with NAFLD-related metabolic pathologies are unsatisfactory [32]. In line with this, exercise has been proposed as an effective treatment strategy for NAFLD through various mechanisms, such as the upregulation of antioxidant enzymes and anti-inflammatory mediators and through the regulation of the endoplasmic reticulum stress-associated pathways [33, 34]. In light of these studies, we identified the HFD-induced NAFLD-linked protein expression profile in sedentary and exercise intervention mouse models through a high-throughput quantification of proteins.

Firstly, we found a significantly increased body weight in HFD group (P < 0.001, Fig 1A), but VWR prevented the weight gain in HFX group (P < 0.001). The food intake was decreased over time in HFD group (P < 0.001), but the normalization of the body weights yielded a significantly increased food intake in HFX group (P < 0.001). In addition, the cumulative food intake of mice was significantly different among groups over time (P < 0.001, Fig 1D). Our results were supported by a recent study which demonstrated that VWR (for 30 min, 5 days) reduces weight gain by preferentially decreasing the intake of high-fat food [35]. Our data showed that HFX group consumed the lowest cumulative food intake among all groups, corroborating with the aforementioned report.

Next, we analyzed the quantitative protein expression profile among groups and established that the level of enolase 1B was higher in the HFD group than in the CON group. Enolase is a glycolytic enzyme which plays an important role of catalyzing 2-phosphoglycerate dehydration to phosphoenolpyruvate as part of glycolytic and gluconeogenesis pathway [36]. Enolase 1 (enolase alpha) is known as a diagnostic marker of multiple tumors and numerous autoimmune diseases [37], including rheumatoid arthritis [38]. A recent study demonstrated that enolase1/MBP-1, functions as a tumor suppressor by binding and inhibiting *c-myc* promoter-binding protein and could play the role of an important sensor/regulator in stressful conditions [39, 40]. In addition, upregulation of enolase 3 in human liver cell line could promote the lipid accumulation. The higher levels of enolase 1B in HFD group suggested that 12 weeks of HFD intake might induce an excessive fat accumulation and cause inflammation and damage to the liver [41]. Our results indicated that the majority of the differentially expressed proteins in the livers of HFD and HFX mice were involved in the energy metabolism of carbohydrates and lipids, as reported previously [42, 43]. Fbp1, a rate-limiting enzyme in gluconeogenesis, is involved in regulating the hydrolysis of fructose 1,6-bisphosphate to fructose 6-phosphate [44, 45]. The accelerated energy expenditure by regular exercise maintains glucose homeostasis in working skeletal muscles through increased glucose uptake and utilization of lipids and muscle glycogen [46]. During prolonged exercise periods such as VWR, the requirement of glucose uptake by contracting muscles is supported by energy homeostasis in the form of glucose release from the liver [47]. Our data demonstrated an increase in liver Fbp1 protein expression after VWR in the HFX group compared with the HFD group. This finding was consistent with previous studies showing that regular exercise could activate metabolic pathways, such as gluconeogenesis, by the oxidation of fatty acids by the liver to fulfill the energy demand. Furthermore, the data suggested that increased gluconeogenesis reflects the increased mitochondrial redox state. Atp5b and mt-Atp8 are mitochondrial membrane ATP synthases that produce ATP from ADP in a proton gradient across the membrane. In our experiments, the mitochondrial precursor-related proteins Atp5b and mt-Atp8 were reduced by HFD, but HFX reversed this effect. In addition, we provided evidence that mitochondrial biogenesis is increased in the HFX group.

The essential proteins that control mitochondrial biogenesis, such as cytochrome b-c1 complex subunit 2, isovalertl-CoA dehydrogenase, and hydroxymethyglutaryl-CoA synthase, were all upregulated in the liver of the HFX mice, compared with the HFD mice. Some previous studies have suggested that exercise improves the function of mitochondrial biogenesis even though the mice were fed an HFD. Lezi et al. observed that 6 weeks of moderate-intensity treadmill exercise increased mitochondrial biogenesis [48]. Gehrke et al. demonstrated that VWR increased mitochondrial fatty acid β–oxidation in the liver of mice fed a HFD for 12 weeks [49]. These findings suggested that HFD decreases energy metabolism and the expression of mitochondrial precursor-related proteins, but exercise reverses these effects in the liver.

Specifically, several essential pathways, including those related to the metabolism of xenobiotics and lipids, were downregulated, resulting in a decrease in SBP2 and Phyh (*LN1*) protein levels in HFD-fed mice. SBP2 is expressed in the liver and has specific properties of binding with xenobiotics like selenium and acetaminophen [50, 51]. Zhou et al. demonstrated that the high fat- and fast food-fed mice groups showed downregulated pathways of carbohydrate/lipid metabolism, including the downregulated expression of *Selenbp2*, but exercise could alleviate the altered DNA methylation induced by the high-fat or fast-food diet [52]. In addition, our experimental results showed that SBP2 levels were increased in HFX mice, corroborating the findings of previous studies. However, the exact role of SBP2 in liver pathology is unclear, but its alteration in response to exercise can make it a novel therapeutic target in various liver diseases. Exercise improves lipid metabolism in the liver. Phyh (LN1) is known to convert phytanoyl-CoA to 2-hydroxyphytanoly-CoA, which is a process of lipid metabolism, and activate β-oxidation [53]. Our study indicated that 12 weeks of VWR increased the expression of the lipid metabolism-related proteins Fbp1, Atp5b, and mt-Atp8.

Aerobic organisms inevitably produce reactive oxygen species (ROS), which are byproducts of oxidative metabolism that could induce oxidative damage to cells [54]. In general, a cell has antioxidant enzymes that protect against the detrimental effects of ROS [54]. However, disrupting the ROS-antioxidant balance under physiological conditions leads to excessive ROS production and the prevalence of metabolic diseases [55]. Our results showed that exercise increased the expression of the liver antioxidant enzymes and proteolysis proteins. Glutathione S-transferase (GST) is a glutathione sulfur transferase enzyme essential for metabolizing pro-oxidant xenobiotics in the liver [54]. There is some evidence that endurance exercise can upregulate liver GST levels, but HFD can downregulate its expression in the liver. Our data showed an increased expression of liver GST P1, 1GUK_A, and Ceb3b after 12 weeks of VWR, suggesting that regular exercise, even with HFD, can increase the levels of antioxidant enzymes in the liver. Regular exercise may protect against excessive alterations in metabolism-related proteins and mitochondrial oxidative proteins.

In summary, in this study we were able to (1) screen the expression profiles of specific proteins in the liver by using MS/MS, (2) verify the functional role of these proteins in our experimental setup, and (3) understand the beneficial effects of exercise in specific pathophysiological conditions. The establishment of high fat diet-induced obesity mouse model is more challenging as a metabolic disease model than the genetic model of obesity, but it may closely resemble the environmental effects of human obesity. Various metabolic conditions, such as metabolic syndrome and NAFLD, can be confirmed by the protein spectrum associated with the disease, as implied by this study. Further understanding of the expression of proteins, such as changes in de *novo* protein synthesis in the liver through exercise, is needed to identify specific protein isoforms to understand mitochondrial bioenergetics under different conditions. The function of unknown proteins (e.g., MCG8752 and MCG129115) found in the current study can be explored in the context of NAFLD and exercise. The limitation of this study was that VWR assumed considerable changes in the expression of proteins, but we did not know the intensity and volume of exercise affecting protein expression in the liver. In addition, if the blood analysis, such as glucose, triglycerides, AST, and so on, was presented together with the current results, it would have supported the results.

In conclusion, in the present study we demonstrated that high-fat diet and regular exercise can modulate proteomics in the liver. The beneficial effects of voluntary wheel running in HFD-fed mice were reflected by the upregulation of gluconeogenesis, detoxification, mitochondrial biogenesis, and proteolysis pathways in the liver.

## Supporting information

S1 Fig Distribution of weekly running distance of mouse in HFX group. In the middle of the box, the cross (+) indicates mean data.
(TIF)
