# Supplementary figures and images for: Proteomic analysis of the effect of high-fat-diet and voluntary physical activity on mouse liver
Source: PLoS One. 2022 Aug 18;17(8):e0273049. doi: 10.1371/journal.pone.0273049 (PMC9387828; doi:10.1371/journal.pone.0273049)

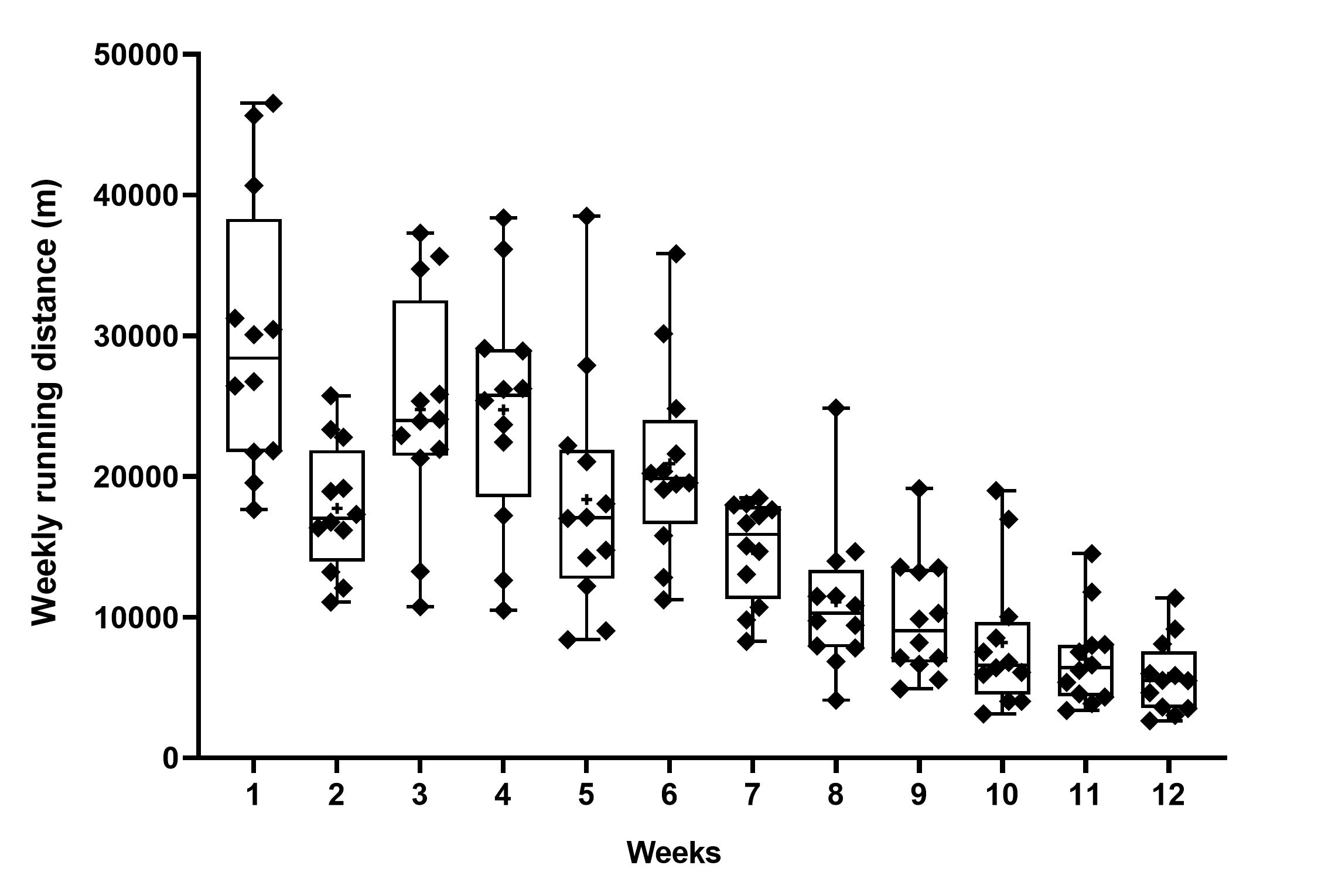

Supplement: S1 Fig — In the middle of the box, the cross (+) indicates mean data. (TIF) [file pone.0273049.s001.tif]
